# Seasonal Differences in Density But Similar Competitive Impact of *Aedes albopictus* (Skuse) on *Aedes aegypti* (L.) in Rio de Janeiro, Brazil

**DOI:** 10.1371/journal.pone.0157120

**Published:** 2016-06-20

**Authors:** Daniel Cardoso Portela Camara, Claudia Torres Codeço, Steven A. Juliano, L. Philip Lounibos, Thais Irene Souza Riback, Glaucio Rocha Pereira, Nildimar Alves Honorio

**Affiliations:** 1 Laboratório de Transmissores de Hematozoários – LATHEMA, Instituto Oswaldo Cruz, Fundação Oswaldo Cruz, Rio de Janeiro, Brazil; 2 Núcleo Operacional Sentinela de Mosquitos Vetores – NOSMOVE, DIRAC – IOC – VPAAPS, Fundação Oswaldo Cruz, Rio de Janeiro, Brazil; 3 Programa de Computação Científica, Fundação Oswaldo Cruz, Rio de Janeiro, Brazil; 4 School of Biological Sciences, Behavior, Ecology, Evolution and Systematics Section, Illinois State University, Normal Illinois, United States of America; 5 Florida Medical Entomology Laboratory, University of Florida, Vero Beach Florida, United States of America; Cary Institute of Ecosystem Studies, UNITED STATES

## Abstract

Previous studies have shown that the negative effects of density of *Ae*. *albopictus* on *Ae*. *aegypti* exceed those of *Ae*. *aegypti* on *Ae*. *albopictus* for population growth, adult size, survivorship, and developmental rate. This competitive superiority has been invoked to explain the displacement of *Ae*. *aegypti* by *Ae*. *albopictus* in the southeastern USA. In Brazil, these species coexist in many vegetated suburban and rural areas. We investigated a related, but less-well-studied question: do effects of *Ae*. *albopictus* on *Ae*. *aegypti* larval development and survival occur under field conditions at realistic densities across multiple seasons in Brazil? We conducted additive competition experiments in a vegetated area of Rio de Janeiro where these species coexist. We tested the hypothesis that *Ae*. *aegypti* (the focal species, at a fixed density) suffers negative effects on development and survivorship across a gradient of increasing densities of *Ae*. *albopictus* (the associate species) in three seasons. The results showed statistically significant effects of both season and larval density on *Ae*. *aegypti* survivorship, and significant effects of season on development rate, with no significant season-density interactions. Densities of *Aedes* larvae in these habitats differed among seasons by a factor of up to 7x. Overall, Spring was the most favorable season for *Ae*. *aegypti* survivorship and development. Results showed that under natural conditions the negative competitive effects of *Ae*. *albopictus* on *Ae*. *aegypti* were expressed primarily as lower survivorship. Coexistence between *Ae*. *aegypti* and *Ae*. *albopictus* in vegetated areas is likely affected by seasonal environmental differences, such as detrital resource levels or egg desiccation, which can influence competition between these species. Interactions between these *Aedes* are important in Brazil, where both species are well established and widely distributed and vector dengue, Zika and chikungunya viruses.

## Introduction

Biological invasions are complex processes, and for an invasive species to become established, it must be capable of increasing in the resident community of the invaded environment. The success of a biological invasion depends on the introduction, establishment, and spread of the invasive species [1, 2]. Typically, such processes affect native species and ecosystems, but may also impact human activities and health [3].

Originally a feral species in Africa, domesticated forms of the yellow fever mosquito *Aedes aegypti* (L.) invaded the New World between the 15^th^ and 17^th^ centuries, becoming one of the most common species found in association with humans [4, 5]. *Aedes aegypti* also successfully invaded Asia in the 19^th^ century, where its increases in abundance were associated with decreases in the abundance of native *Aedes albopictus* (Skuse), in Calcutta and other southeast Asian cities [6, 7]. *Aedes albopictus*, native to Asia, has successfully invaded and established in the Americas, Europe, and Africa, mostly in the past three decades, including expansions into temperate regions [7, 8]. The introduction, establishment and spread of this species made it common in artificial containers throughout the southeastern United States, where it frequently displaced resident *Ae*. *aegypti* [9, 10]. However, *Ae*. *aegypti* still remains common in urban south Florida, and a few other cities of the southeastern United States [9], frequently coexisting with *Ae*. *albopictus* [11, 12].

In Brazil, *Ae*. *albopictus* was first detected in 1986 and, although its spread and establishment were not continuously monitored, by 2014 *Ae*. *albopictus* was detected in 25 of the 27 Brazilian states, and in 59% of Brazilian municipalities [13, 14, 15]. This makes it a common species in artificial containers in suburban and urban areas of Brazil, often co-occurring with *Ae*. *aegypti* [11, 16, 17]. *Aedes aegypti* remains more common in urban areas whereas *Ae*. *albopictus* favors suburban and rural vegetated areas in Brazil [11, 18].

Both species share similar larval habits, including development in water-holding artificial containers. As a result of their overlapping geographic distributions and shared microhabitats, invasions by these species have impacted the distribution and abundance of one another, as well as of other resident mosquito species [2, 7, 9]. It has been proposed that interspecific competition during larval development contributed to displacements of *Ae*. *aegypti* by *Ae*. *albopictus* in the Americas. Evidence for this hypothesis comes from laboratory [19, 20] and field experiments in the USA [21, 22, 12] and Brazil [22], demonstrating that *Ae*. *albopictus* is the superior competitor especially in resource-limited conditions [19, 20, 21, 23].

However, the outcome of competition between these species is highly context-dependent and affected by the nature of aquatic resources (as reviewed by [24]). Outcomes can be altered by detritus type, shifting a situation of intense competition to a state of low interspecific competition and stable coexistence between *Ae*. *albopictus* and *Ae*. *aegypti* [23]. A diet based on rapidly decaying resources (e.g., yeast, animal detritus, such as dead insects and liver powder) can reduce the competitive advantage of *Ae*. *albopictus* [19, 20, 25, 26] allowing coexistence while a diet based on deciduous or coniferous leaves tends to accentuate the competitive advantage of *Ae*. *albopictus*.

Controlled competition experiments have shown evidence of negative effects of *Ae*. *albopictus* larvae on the adult size, survival to adulthood, development rate, and adult longevity of *Ae*. *aegypti* [21, 22, 27, 28]. Although the populations of *Ae*. *albopictus* that invaded both the United States and Brazil have different geographic origins [29], a previous study suggests that *Ae*. *albopictus* is a superior competitor to *Ae*. *aegypti* in Brazil as well as in the USA [22].

In subtropical Florida, mainly characterized by a subtropical climate, with warm temperatures and low rainfall in the winter months [30], seasonal differences in quantity and quality of detritus input to containers also seem to alleviate effects of competition in the spring dry season, and may thus also contribute to local coexistence of these species in seasonally variable areas [31]. Indeed, the detritus input in containers in Florida are significant predictors of abundance of *Ae*. *aegypti* in this region, contributing to the distribution of both *Ae*. *aegypti* and *Ae*. *albopictus* [32]. Differences in seasonal climate also appear to affect the distribution and coexistence of these species [33], and desiccation can alter the competitive balance between these species in the laboratory, via differential mortality on *Ae*. *albopictus* eggs [34].

This study tests the hypothesis that interspecific competition from *Ae*. *albopictus* impacts *Ae*. *aegypti* under variable field conditions, which change seasonally in the tropical climate of southeastern coastal Brazil. Although seasonal climate changes are not markedly strong in the region [35], the abundance of *Ae*. *aegypti* and *Ae*. *albopictus* fluctuates seasonally with a peak during the wet season [18]. We quantified how *Ae*. *aegypti* (the focal species) responds to *Ae*. *albopictus* (the associate species) abundance by manipulating larval densities of *Ae*. *albopictus* across a range typically observed in the field. Based on previous field studies [12, 21, 22, 30], we predicted that increasing densities of *Ae*. *albopictus* would negatively affect survivorship and development of *Ae*. *aegypti* and that this impact would vary with season. These additive experiments are expected to enhance our understanding of how these species interact and coexist in Brazil, where they may transmit either dengue or chikungunya viruses.

## Materials and Methods

### Study area

**Fig 1:**
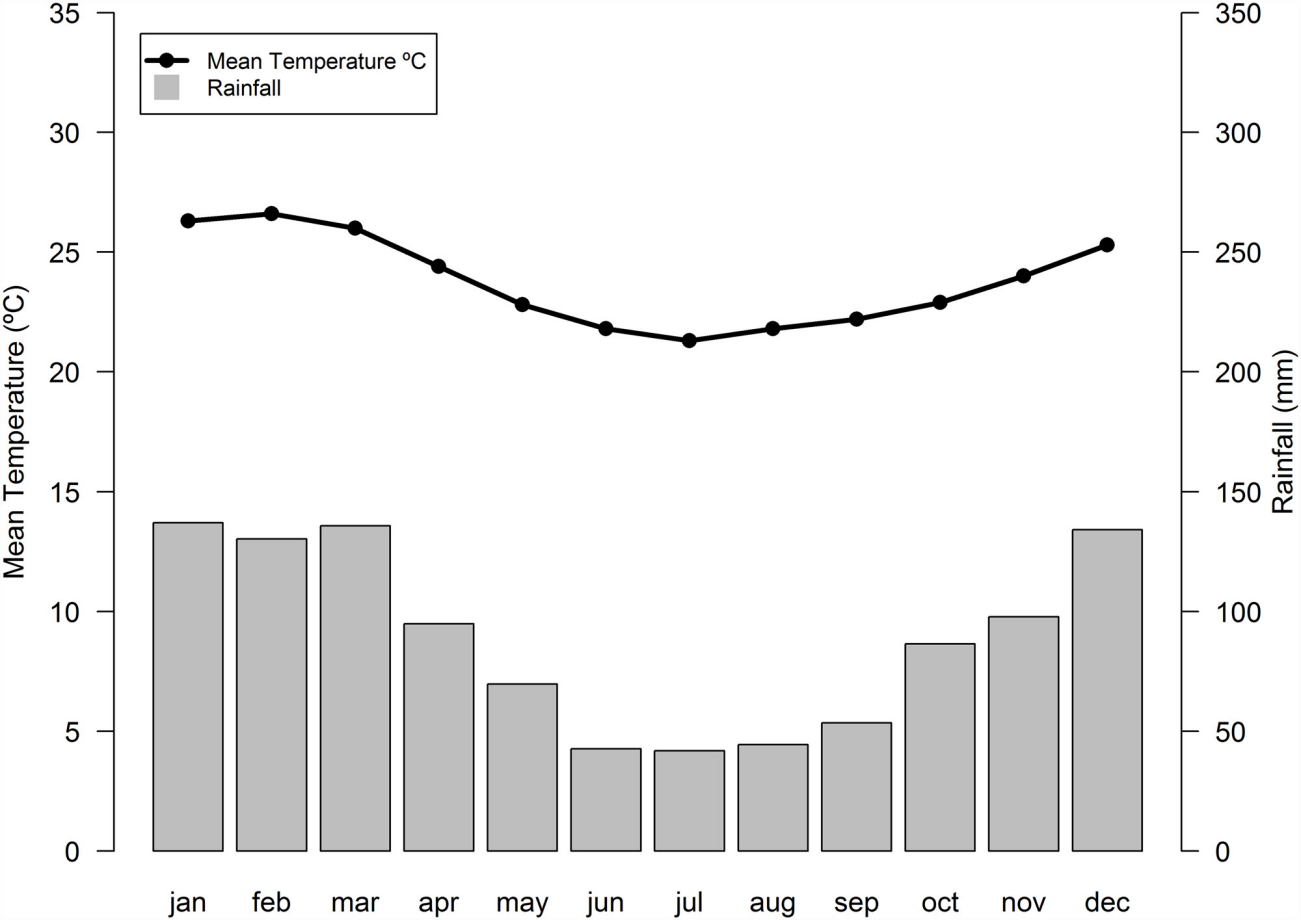
Climate normals (1961–1990) for the city of Rio de Janeiro. Instituto Nacional de Meteorologia—Brazilian National Institute of Meteorology, available at http://www.inmet.gov.br/.

The experiments were done in the botanical garden of Fundação Oswaldo Cruz—FIOCRUZ (Oswaldo Cruz Foundation, 22°5’S, 43°2’W), in Rio de Janeiro on a campus of 9 km^2^ where *Ae*. *aegypti* and *Ae*. *albopictus* co-occur [18]. The FIOCRUZ campus is surrounded by densely populated slums (favelas), where *Ae*. *aegypti* abundance is high, piped water is irregular, and garbage removal is deficient [18, 36]. The botanical garden of FIOCRUZ is a secondary patch of Atlantic Rain Forest, with constant litterfall throughout the year, as observed elsewhere in the same biome [37]. The experiments were done in the Autumn (May—June) and Spring (September—October) of 2011 and in the Summer (January—February) of 2012. Mean temperature varies between 20°C and 27°C, and monthly rainfall varies between 41 mm and 137 mm. Summer and Autumn are typically wet, with rain being most frequent from December to March, although rain occurs throughout the whole year [35] (Fig 1).

#### Meteorological data

Meteorological data used to describe environmental conditions during the experiments were obtained from BDMEP—Banco de Dados Meteorológicos para Ensino e Pesquisa (Meteorological Database for Teaching and Research, available at http://www.inmet.gov.br/projetos/rede/pesquisa/). The data used spanned the 30 days of the colonization periods in each experiment. The meteorological station is situated approximately 5 km from the study area.

### Experiments

Each of the three experiments consisted of two phases: a 30-day colonization phase, followed by a 7-day experimental phase.

#### Colonization phase

For each experiment, we randomly distributed 45 small black plastic vases (with maximum capacity of 3L) in shaded or partially shaded locations within the botanical garden, with individual vases at least 3 meters apart. Each vase was filled with 1L of distilled water and secured to a wooden stake. The vases remained in the garden for 30 days, with water (as rainfall) and resource input occurring naturally, the latter as fallen leaves, fruits, seeds, dirt and invertebrates that accumulated during this period. Each vase was inspected daily, and all mosquito pupae were collected and taken to the Núcleo Operacional Sentinela de Mosquitos Vetores—NOSMOVE/Fiocruz, to prevent vector emergence in a dengue endemic area. In the laboratory, each pupa was confined in an individual container and kept in an incubator at 27°C ± 1°C until adult emergence. Adults were killed by freezing, sexed and identified to species.

On day 30, when the colonization period was complete, the contents of each vase were removed. Larvae and pupae were transported in 50 mL Falcon tubes to the laboratory for identification and counting. The remaining water and detritus from each vase was transferred to a new identical vase, which was covered with 0.5 mm nylon mesh secured with a rubber band to prevent further oviposition from wild mosquitoes.

The number of mosquito pupae collected during the colonization period and the number of larvae present in the last day of colonization were summed and divided by the initial number of vases to obtain a mean baseline immature density (rounded to the nearest 10 larvae; symbolized hereafter as B) to be used in competition experiments. In each season, the mean baseline immature density (B) was considered as an estimate of the natural density (Table 1). As mosquito productivity in vases varied among seasons, seasonal baseline mean numbers also varied accordingly.

**Table 1:** Larval and pupal productivity during the colonization period defined the Baseline number for the larval manipulation experiments.

Season	# vases (n)	# of Productive vases	Total pupae + larvae collected during colonization (T)	*Ae*. *aegypti* proportion in the colonization period	Baseline Number B = T/n
Autumn	45	41	593 + 2670	0.007	70
Spring	45	39	853 + 702	0.527	40
Summer	45	43	4413 + 8414	0.047	290

#### Competition phase

In each season, an addition series competition experiment [38] was conducted with three density treatments and 15 replicates. The treatments consisted of a fixed number of *Ae*. *aegypti* (the focal species) first instar larvae (0.5 B, the Baseline number B) plus one of three densities of *Ae*. *albopictus* (the associate species), defining three total density levels: The LOW treatment consisted of 0.5 B *Ae*. *aegypti* and no *Ae*. *albopictus*, defining a low density. The FIELD treatment had 0.5 B *Ae*. *aegypti* and 0.5 B *Ae*. *albopictus*, resulting in a crowding level similar to that of the natural mean density for that season. The HIGH treatment consisted of 0.5 B of *Ae*. *aegypti* and 1 B of *Ae*. *albopictus*, resulting in a total density 1.5x greater than the baseline number and defining a high crowding environment (Table 2). Thus, larval density in the HIGH treatment was 3x that of the LOW treatment. A similar approach was used successfully in field manipulations of vases testing for competitive effects of *Ae*. *albopictus* on *Ae*. *aegypti* in Florida cemeteries [12]. Addition series are designed to quantify whether the effect of the associate species (*Ae*. *albopictus*) on the focal species (*Ae*. *aegypti*) is different under different seasonal conditions [38].

**Table 2:** Number of 1^st^ instar larvae of *Ae*. *aegypti* and *Ae*. *albopictus* used in each density treatment in the three experiments.

Season	LOW density		FIELD density		HIGH density	
	*Ae*. *aegypti*	*Ae*. *albopictus*	*Ae*. *aegypti*	*Ae*. *albopictus*	*Ae*. *aegypti*	*Ae*. *albopictus*
Autumn	35	0	35	35	35	70
Spring	20	0	20	20	20	40
Summer	145	0	145	145	145	290

Experimental larvae were hatched from eggs harvested from open colonies maintained by the Laboratório de Transmissores de Hematozoários (LATHEMA/IOC—FIOCRUZ), established from specimens collected in Rio de Janeiro. Around 5000 eggs of each species were hatched in plastic bowls with 1 L of distilled water and 1 g of Tetramin^®^ fish food. Approximately 24 hours after hatching, larvae were counted and added to the vases in the field, in the appropriate numbers for the different density treatments. The experiment ended on the seventh day, when all vases were carefully inspected. No adults were found in any of the three experiments, and all larvae and pupae were collected and brought to the laboratory in sealed, 500 mL Whirl Pak bags. All individuals were identified by species and instar.

### Statistical analysis

All analyses were done using R 3.1.0 [39] and RStudio [40], with the “car” package [41], at a significance level of 5%. Seasonal differences in mean temperature were assessed by one-way ANOVA and rainfall by a Kruskal-Wallis test. Heterogeneity of rainy days (rainfall ≥ 1mm) among experiments was assessed by Chi-square test. *Aedes aegypti* and *Ae*. *albopictus* pupal productivity during the colonization period was compared between species and seasons using a Kruskal-Wallis test, followed by multiple Mann-Whitney tests with Bonferroni corrections. The effect of *Ae*. *albopictus* density on *Ae*. *aegypti* performance was measured via two life-history parameters: (1) survivorship, defined as the proportion of *Ae*. *aegypti* larvae in each container that survived to the end of the experiment at 7 days and (2) mean instar, defined as the sum of instar codes for all immatures present at the end of the 7-day period (numerical coding as larval instars = 1 to 4, pupa = 5), divided by the number of survivors. A two-way ANOVA was performed, with season (Autumn, Spring, and Summer) and treatment (LOW, FIELD, and HIGH) as fixed factors. The Box-Cox procedure was used to analyze both ANOVA results to verify the need for data transformation. We squared the *Ae*. *aegypti* survivorship so that the residuals fitted a normal distribution.

## Results

### Climate

**Fig 2:**
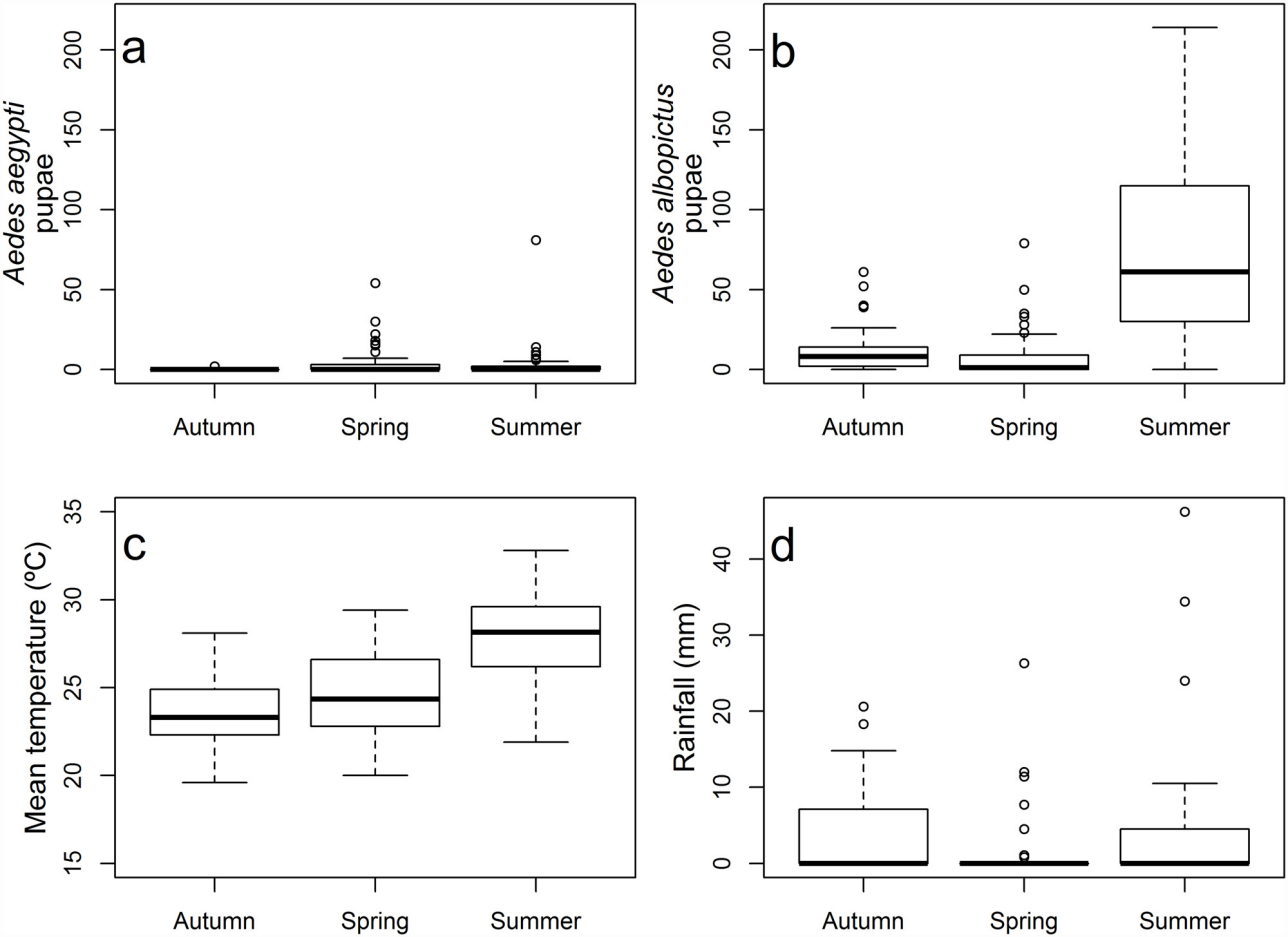
Pupal productivity and Climate data during the colonization period. Pupal productivity of *Ae*. *aegypti* (a) and *Ae*. *albopictus* (b), mean daily temperature (c) and daily rainfall (d) during the 30-day period of colonization in the Autumn, Spring and Summer.

The weather during the experiments was typical for these months of the year. Air humidity exceeded 60% during the whole study, except in four summer days and one spring day. Air temperature varied from 17.2°C (spring) to 39°C (summer). Minimum temperatures, that could affect larval development, were similar during the Autumn and Spring months, varying from 17.3 to 23.5°C in the former and 17.2 to 24.0°C in the latter. Summer was significantly warmer (mean temperature ± SE, 27.88 ± 0.49°C) than Autumn (23.41 ± 0.36°C) and Spring (24.52 ± 0.44°C) (F_2,87_ = 28.89, p < 0.001) (Fig 2c). Total precipitation during the colonization experiment was higher in the Summer (144.2 mm), followed by the Fall (117.1 mm) and Spring (63.8mm). This precipitation spread through 14, 13, and 7 days of the 30 experiment days in each season, respectively. Daily rainfall did not differ significantly between seasons, with an average of 9 to 10 mm (Kruskal-Wallis Chi-square = 3.3929, df = 2, p-value = 0.1833), but the summer experienced two storms, of 34.4 mm and 46.2 mm; these values exceed the maximum precipitations observed in the Spring and Autumn experiments (26 and 20 mm, respectively).

### Colonization phase

The 30 days colonization experiments produced 648 pupae of mosquitos in the Autumn, 900 in the Spring and 4765 in the Summer. Pupae of *Ae*. *aegypti*, *Ae*. *albopictus* and *Limatus durhami* were found in all seasons, while *Aedes fluviatilis* was present only in the Spring and Summer and *Ae*. *scapularis* only in the Autumn and Spring (Table 3). Daily pupal productivity was significantly different between seasons (Kruskal-Wallis Chi-square = 58.7552, df = 2, p < 0.0001) and post-hoc tests indicate that summer was the cause of this difference.

**Table 3:** Relative abundance of the pupal productivity for each mosquito species and mean (standard error) pupae produced per vase at the end of the 30 days period of colonization in the Autumn, Spring and Summer.

	Autumn		Spring		Summer	
Species	Relative abundance	Mean (SE)	Relative abundance	Mean (SE)	Relative abundance	Mean (SE)
*Aedes aegypti*	0.007	0.089 (0.062)	0.224	4.244 (1.511)	0.037	3.622 (1.828)
*Aedes albopictus*	0.885	11.667 (2.084)	0.424	8.044 (2.332)	0.775	76.044 (8.718)
*Aedes scapularis*	0.051	0.667 (0.473)	0.130	2.467 (2.467)	-	-
*Aedes fluviatilis*	-	-	0.175	3.311 (1.752)	0.022	2.111 (1.177)
*Limatus durhami*	0.057	0.756 (0.497)	0.047	0.889 (0.541)	0.166	16.289 (2.713)

*Aedes aegypti* pupal productivity was significantly greater in the Spring (n = 191) and Summer (n = 163) than in the Autumn (n = 4) (Fig 2a, Table 4). *Aedes albopictus*, on the other hand, reached the highest production in the Summer (n = 3422 pupae), followed Autumn (n = 525) and by Spring (n = 362) (Fig 2b). *Aedes albopictus* was more productive than *Ae*. *aegypti* in all seasons, although this difference was only marginally significant in the Spring (Table 4). Together, these two species dominated the container mosquito community, with 89.2, 64.8 and 81.2% of the pupae found in the Autumn, Spring and Summer experiments, respectively (Table 3).

**Table 4:** Kruskal-Wallis test for significant differences in pupal productivity between Autumn, Spring and Summer colonization period, and Mann-Whitney pairwise comparisons with Bonferroni correction. Mann-Whitney tests were used for comparisons between *Ae*. *aegypti* and *Ae*. *albopictus* (overall and in each season).

Pupal productivity	Seasons	Autumn x Spring	Autumn x Summer	Spring x Summer
*Ae*. *aegypti*	**KW = 17.4895, df = 2, p < 0.001**	**p-value < 0.01**	**p-value < 0.001**	p-value = 1
*Ae*. *albopictus*	**KW = 57.0234, df = 2, p < 0.001**	**p-value < 0.05**	**p-value < 0.001**	**p-value < 0.001**
*Ae*. *aegypti x Ae*. *albopictus*	**W = 3643, p < 0.001**	**W = 217.5, p-value < 0.001**	W = 812.5, p = 0.07499	**W = 125, p-value < 0.001**

KW = Kruskal-Wallis test statistics; df = degrees of freedom, W = Wilcoxon rank sum test statistics.

### Competition phase

#### Mean instar

**Fig 3:**
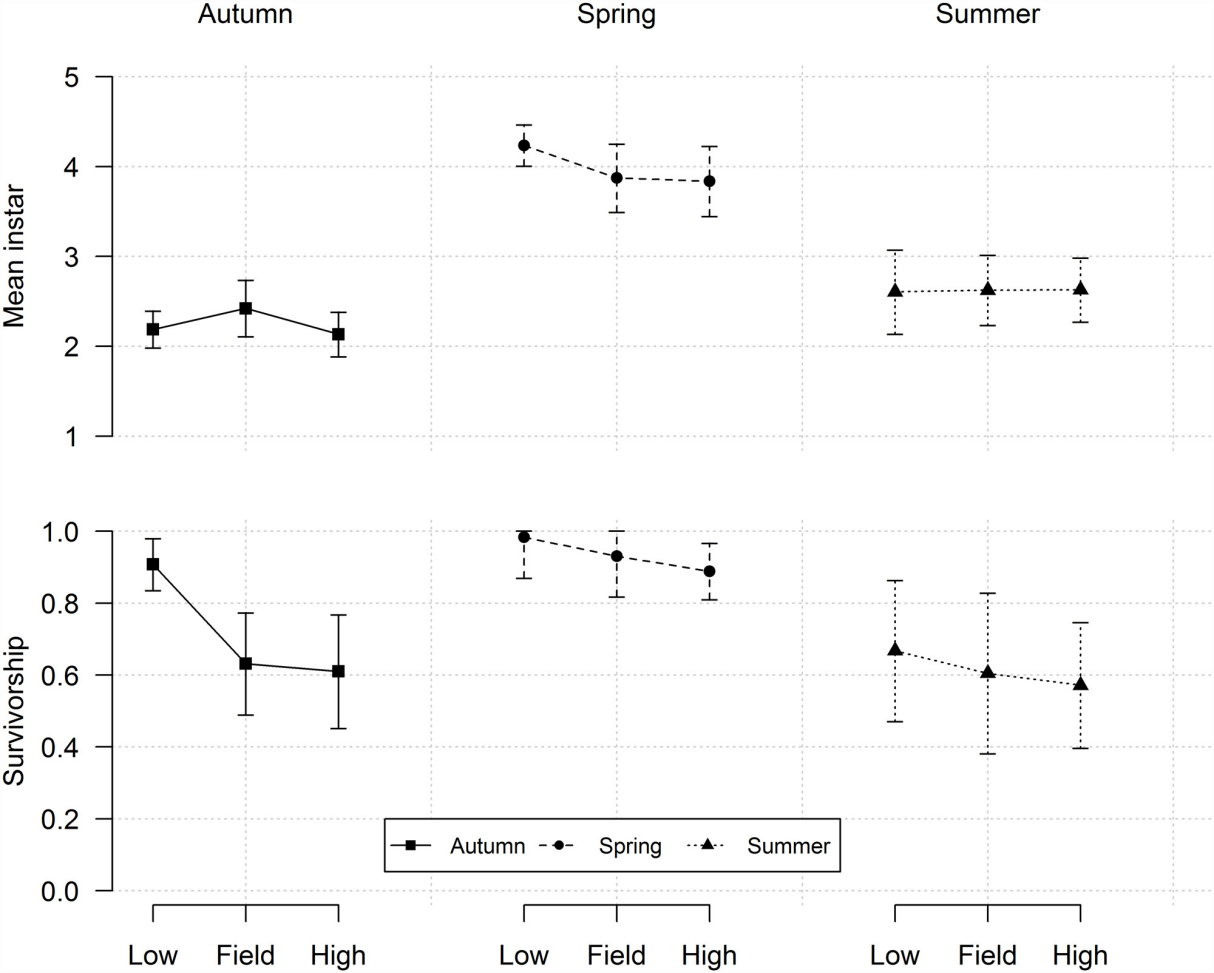
Competition experiment results. Mean (± 2 SE) for competition treatments for mean instar of *Ae*. *aegypti* and survivorship after 7 days of experiment in the Autumn, Spring and Summer.

We found no significant effect of *Ae*. *albopictus* density on *Ae*. *aegypti* mean instar (Table 5, Fig 3). Season, on the other hand, significantly affected mean instar (Table 5). In the Spring, after 7 days, all larvae had already passed through the third instar and most were in the fourth instar independent of the presence or density of *Ae*. *albopictus* (Table 5, Fig 3). In contrast, in the Summer and Autumn, most larvae were still second instars, with no clear difference between these seasons.

**Table 5:** Two-way ANOVA for the effect of *Aedes albopictus* density and season on the mean larval survivorship and developmental progress of *Aedes aegypti* after 7 days (mean instar).

Response	Survivorship			Mean Instar		
	DF	F	P	DF	F	P
Density	**2**	**5.3486**	**0.0062**	2	0.8146	0.4457
Season	**2**	**16.1776**	**< 0.0001**	**2**	**101.5853**	**< 0.0001**
Density x Season	4	1.9179	0.1133	4	1.1405	0.3419
Error	100	100		100		

#### Survivorship

Both *Ae*. *albopictus* density and season had significant effects on the survivorship of *Ae*. *aegypti* (Table 5). At field density, survival was higher in the Spring (>90%), compared to the 60–65% survival in the other seasons. As density was relaxed, survival improved in all seasons, particularly in the Autumn (90% survival). In the Summer, this density effect is small. As density increases above field levels, survival slightly decreases in all seasons (Fig 3).

## Discussion

This study tested the hypothesis that *Ae*. *albopictus* negatively affects *Ae*. *aegypti's* life history under field conditions in Rio de Janeiro. Such negative effects may have important consequences on the vectorial capacity of *Ae*. *aegypti* [25, 28, 42, 43], and thus are of potentially great public health importance, as Rio de Janeiro is a dengue endemic area, suffering from frequent epidemics [44, 45]. Our results have shown only seasonal effects on development rate, whereas both *Ae*. *albopictus* density and season affected larval survivorship (Table 5). Thus, we find evidence that *Ae*. *aegypti* suffers negative competitive effects from *Ae*. *albopictus* under these natural conditions. These effects are manifest primarily as lower survivorship, rather than delayed development. Moreover, the lack of interaction between density and season suggests that the effects of competition are similar across seasons.

Seasonality in Rio de Janeiro is relatively mild, and climate is mostly within the range considered favorable for *Aedes* development [4, 7]. Still, our results suggest this seasonal variation is sufficient to impact on the productivity of mosquito breeding sites. The least productive season was the Spring, with an average of 40 larvae per container; in this season the temperature was similar to the Autumn and the precipitation was low. Autumn was moderately productive, with 70 larvae per container, and Summer was the most productive season (Table 1). Differences in the total number of immatures observed between Spring and Autumn, which presented similar weather conditions could be due to differences in standing crop of adults in the area (Table 1).

We found significant density effects of *Ae albopictus* on *Ae*. *aegypti* larval survivorship but not on their development rate in all seasons but the effect on survivorship appeared to be stronger in the Autumn. In the Autumn, relaxing the density of *Ae*. *albopictus* improved *Ae*. *aegypti* survivorship from 60% to 90%. In the Spring survivorship was high at all densities and in the Summer it was low at all densities. These are important results, showing that the negative effects of *Ae*. *albopictus* on *Ae*. *aegypti* vary under natural conditions.

Many studies have shown that *Ae*. *aegypti* and *Ae*. *albopictus* coexist in Brazil. Both species are abundant in Rio de Janeiro with *Ae*. *aegypti* predominating inside highly urbanized areas and *Ae*. *albopictus* in more rural areas [11]. An important finding was that *Ae*. *aegypti* was abundant in forest edges inside Rio de Janeiro [11]. *Aedes aegypti* coexisted with *Ae*. *albopictus* in transition areas between highly urbanized and highly vegetated areas in the same study area in which our experiments were performed [18]. In suburban areas *Ae*. *aegypti* and *Ae*. *albopictus* coexist in high numbers [17, 46]. Moreover, in the transition area between urban and sylvatic environments of the largest urban forest of Rio de Janeiro, *Ae*. *aegypti* favored oviposition in containers inside and near houses [47], and a low tendency to disperse into the forest, colonizing traps only up to 100 m inside the forest [48]. Together, these studies show that both species are widespread and common in Rio de Janeiro, coexisting in many suburban areas, but also in transition zones. Although our results show that *Ae*. *albopictus* predominates containers in the study area (Tables 1 and 3), *Ae*. *aegypti* was successful in colonizing vases during the colonization phase, particularly in Spring and Summer. Our results show not only the complex and seasonal dynamics of *Aedes* species, which are capable of colonizing artificial containers inside forested areas, but also that these species are under constant competitive stress.

Although the competitive superiority of *Ae*. *albopictus* on *Ae*. *aegypti* has been observed in both field [21, 22] and laboratory experiments [19, 20], *Ae*. *aegypti* still persists in urban south Florida [9, 49, 50]. Field experiments carried out in Florida, using approximately natural larval densities in cemetery vases have shown that interspecific competition between *Ae*. *aegypti* and *Ae*. *albopictus* is common in nature [12]. These field experiments were done during the early and late wet season, at sites of coexistence of these *Aedes* species and at sites where *Ae*. *aegypti* was displaced by *Ae*. *albopictus*, showing that negative competitive effects of *Ae*. *albopictus* on *Ae*. *aegypti* were indistinguishable among these sites. Similar results were found in a forest patch in Florida, demonstrating significantly lower intensity of competition in the dry season compared to the rainy season, when *Ae*. *albopictus* had a competitive advantage [31].

A field experiment in Rio de Janeiro using Brazilian *Ae*. *aegypti* and *Ae*. *albopictus* populations quantified performance of larvae of both species at predefined densities that produced interspecific competition [22]. The controlled experiment in [22] was the first to use Brazilian populations of both mosquito species to test for the effects of intra- and inters-pecific competition, and demonstrated the competitive superiority of *Ae*. *albopictus*. Our results provide evidence that the interspecific competitive effect of *Ae*. *albopictus* on *Ae*. *aegypti* is strong in nature and that there are seasonal effects that may contribute to the coexistence of these species in Brazil, adding to an important body of information on the biology and ecology of these species in the country. Although our experiments showed negative competitive effects of *Ae*. *albopictus* on *Ae*. *aegypti*, we had expected that the high baseline numbers in the Summer experiment would yield greater impacts on *Ae*. *aegypti* (Table 1). Our results suggest instead that there may be external factors that were not measured in our experiment that might help to explain the outcome of interspecific competition between these species and the different patterns of coexistence within a single year. Below, we give two possible explanations for this.

One explanation is that seasonal variation in the abundances of *Ae*. *aegypti* and *Ae*. *albopictus*, and ultimately their coexistence in this part of Brazil, may be related to climate, and particularly to the differential ability of these species to survive as dormant eggs during dry periods [33]. Females of both species lay desiccation-tolerant eggs on the sides of containers, which may survive many months [4, 7]. Furthermore, laboratory [33, 51] and field [50] experiments showed that eggs of *Ae*. *aegypti* are more resistant to desiccation than eggs of *Ae*. *albopictus*. In south Florida, where climate is subtropical with strong seasonality in precipitation, *Ae*. *aegypti* not only persisted after the invasion of *Ae*. *albopictus*, but these species coexist in many areas [9, 49, 50]. Rio de Janeiro has a similar pattern of coexistence between *Ae*. *aegypti* and *Ae*. *albopictus* [11, 17, 18, 46], and its tropical climate has strong seasonal patterns of precipitation (Fig 1). Indeed, the drying of containers is much more detrimental to *Ae*. *albopictus* than to *Ae*. *aegypti*, and its effects on noncompeting life stages (causing higher mortality in the eggs of the former species) can alter the outcome of competition between both species, favoring the more desiccation-tolerant *Ae*. *aegypti* [33, 34, 50]. Although our experiment was not designed to test effects of desiccation on egg mortality of both species, there are clear differences on the relative abundance of *Ae*. *aegypti* and *Ae*. *albopictus* in our study. Spring was the most favorable season for *Ae*. *aegypti*, with higher numbers of immatures collected, and the least favorable for *Ae*. *albopictus* (Tables 1 and 3). As the Spring is the end of the dry season in Rio de Janeiro (Fig 1), egg desiccation might be influencing *Ae*. *aegypti* and *Ae*. *albopictus* coexistence.

Another potential explanation for coexistence is based on the detritus accumulation in the vases. Food quality has been shown to alter the outcome of interspecific competition, with rich and rapidly decaying detritus (usually including animal material) reducing the competitive disadvantage of *Ae*. *aegypti*, allowing stable coexistence between these species [19, 20, 24]. The type of detritus used as source of food not only affects survivorship of both species, but also yields different outcomes in competition, with high-quality detritus favoring *Ae*. *aegypti*, and low-quality detritus favoring *Ae*. *albopictus* [23]. Indeed, detritus input in containers is a strong predictor of abundance of both *Aedes* species, and may be responsible for their spatial patterns in Florida. A similar seasonal experiment was done in Florida, demonstrating significant seasonal differences in the intensity of competition, with competitive effects favoring *Ae*. *albopictus* disappearing in the dry season [31]. The authors suggested that the best explanation for their observed seasonal difference in competition’s impact was seasonally different resource inputs to containers, and that this may have equalized fitness differences between the species, prolonging the expected time to competitive exclusion, and ultimately making coexistence more likely than exclusion [31].

In our experiment, however, the lack of interaction between season and density suggests that the impact of competition on *Ae*. *aegypti* is strong throughout the seasons and in each density tested (LOW, FIELD and HIGH), with no seasonal differences (Table 5). Although we have no data on resource input to vases during our experiment, litterfall data from elsewhere in Atlantic rainforest (the biome in which our study took place) shows greatest litterfall in the rainy season, which begins during the Spring [52, 53, 37], which was the season that seemed most favorable for *Ae*. *aegypti*, and least favorable for *Ae*. *albopictus*. The lack of interaction suggests that quality or quantity of seasonal resource input is unlikely to explain fully coexistence between these species. Indeed, literfall in tropical South America seems to be heavily dependent on rainfall seasonality and conservancy of the forest, even though areas of anthropogenic perturbation seem to produce more literfall than areas less perturbated [37]. These results suggest external factors such as mortality induced via egg desiccation contributes to the coexistence of these species [33, 50].

*Aedes albopictus* is now present in almost 60% of the 5,570 Brazilian municipalities, where *Ae*. *aegypti* is also present in many of the same places [14, 15]. Thus, we believe that our results should encourage more laboratory and field studies focused on the interactions between these two species. There are many interesting questions left to be studied regarding Brazilian populations of *Ae*. *aegypti* and *Ae*. *albopictus*, and a focus on how climate (air temperature, drying regime) and the quality and abundance of resources can affect the outcome of interspecific competition. Also, there is a need to understand interspecific interactions between adult populations, like interspecific mating or satyrization, which may be responsible for shaping the distribution of both species. There are evidences of interspecific mating between *Ae*. *aegypti* and *Ae*. *albopictus* under laboratory conditions [54] and on four continents where the two species occur in sympatry [55, 56]. It was also shown that *Ae*. *aegypti* females are more likely to mate with *Ae*. *albopictus* males than the converse interspecific cross [57]. In Brazil, *Ae*. *aegypti* females suffer significant negative effects of cross-insemination, which may play an important role not only in this species distribution, but also on dengue transmission in an endemic area [58]. Even though investigations of interactions between *Ae*. *aegypti* and *Ae*. *albopictus* are prominent in North America, Brazilian populations of both mosquito species are still under-studied [3, 11, 22, 24].

Studies on these vector mosquitos are of particular importance in Brazil, as both are well established and widely distributed in a country where dengue is endemic, and now zika and chikungunya are of major public health concern. All four dengue serotypes are widespread in Brazil, with incidence and proportion of severe cases increasing in the last decade [45]. Recently, it was reported that both the East/Central/South African and Asian chikungunya genotypes are present in Brazil, causing thousands of cases [59]. Moreover, the zika epidemics is ravaging most coastal areas of Brazil, and its relationship with microcephaly in newborns poses as one of the most critical public health problems of the last decades [60]. Since the coexistence of *Ae*. *aegypti* and *Ae*. *albopictus* and the strength of the interspecific competition in larval habitats affect vector competence for arbovirus [25, 42], and Brazilian populations of both species are competent vectors for chikungunya virus [61] and zika virus [62], there is a need to develop further studies focusing interactions of both species in Brazil.

## Supporting Information

S1 File Zip file containing the dataset used in this study. It consists of three.csv files: climate_data.csv contains the climate variables for the study period, competition_data.csv contains the data from the competition experiments, productivity_data.csv contains the total number of pupae collected during the colonization experiment.
(ZIP)
